# Banking on iPSC- Is it Doable and is it Worthwhile

**DOI:** 10.1007/s12015-014-9574-4

**Published:** 2014-12-17

**Authors:** Susan Solomon, Fernando Pitossi, Mahendra S. Rao

**Affiliations:** 1 New York Stem Cell Foundation, 1995 S. Broadway, New York, NY 10023 USA; 2 Q therapeutics, Salt Lake City, UT 84108, New York Stem Cell Foundation, New York, NY 10032 USA; 3 Laboratory for Regenerative and Protective Therapies of the CNS, Institute Leloir Foundation- IIBBA CONICET Patricias Argentinas 435, Buenos Aires, Argentina

**Keywords:** Induced pluripotent stem cells, Embryonic stem cells, Manufacturing, cGMP, Consent, Markers

## Abstract

The discovery of induced pluripotent stem cells (iPSCs) and concurrent development of protocols for their cell-type specific differentiation have revolutionized studies of diseases and raised the possibility that personalized medicine may be achievable. Realizing the full potential of iPSC will require addressing the challenges inherent in obtaining appropriate cells for millions of individuals while meeting the regulatory requirements of delivering therapy and keeping costs affordable. Critical to making PSC based cell therapy widely accessible is determining which mode of cell collection, storage and distribution, will work. In this manuscript we suggest that moderate sized bank where a diverse set of lines carrying different combinations of commonly present HLA alleles are banked and differentiated cells are made available to matched recipients as need dictates may be a solution. We discuss the issues related to developing such a bank and how it could be constructed and propose a bank of selected HLA phenotypes from carefully screened healthy individuals as a solution to delivering personalized medicine.

## Using IPSC for Cell Based Therapy

The ability to harvest somatic cell from any individual and reprogram them with high fidelity and reasonable efficiency has lead to proposal of personalized medicine where either autologous or HLA matched tissue cells could be obtained and then used to make iPSCs that are differentiated into the appropriate required phenotype [1–3]. Several models of such cell based therapy have been proposed [4]. One model is to use autologous PSC-derived cell products or engineered PSC-derived cells for cell replacement or as a vehicle for the delivery of a payload such as an enzyme or drug. Like other autologous cell therapies, the use of patient-specific PSCs will bypass the issue of immune rejection. Alternatively, if HLA matched banks of iPSCs are available, this “hybrid” model will allow the selection and use of optimally-matched cells to produce graft material that will only require limited immune suppression [5–7].

The truly autologous model although ideal in principle suffers from several practical disadvantages. Perhaps the most important one is that it takes time to generate an iPSC line. This time ranges in terms of weeks and months rather than days. Performing the selection and characterization of a clone as required by FDA regulations for all “more than minimally processed cells based therapy” adds additional time to the generation process as well as adding significantly to the cost of therapy. Further autologous cells may carry gene defects that will need to be corrected and thus may require further time to process, characterize and make available to the patient. This may restrict the use of such autologous cells to only chronic diseases where sufficient time is available to perform the necessary processes and the benefits of the therapy are proportionate to the cost of this process. The choice of such autologous therapy may be further restricted if gene engineering is not sufficiently efficient or if the regulations require additional testing of each subclone made [8–10].

A HLA matched bank model obviates some of these issues. A pool of cells can be made based on allelic frequencies of HLA phenotypes and standard HLA matching designs can be used to give individuals a reasonable probability of obtaining a match. Unlike other cells the IPSCs are a virtually infinite supply so such a bank once set up would not be depleted by demand. Current estimates are that a relatively small number (in the hundreds) of lines carefully selected based on allelic frequencies would be sufficient [5–7]. More importantly the effort could be spread worldwide so that each group of individual needed to contribute a small subset of lines making the cost quite manageable. While the initial set-up would be expensive [8, 9] the availability of an off the shelf product that is rigorously tested and widely available would be much easier for the regulatory authorities to grant approval for. Equally important since carefully screened donors are selected that are healthy and do not carry major susceptibility genes one can reduce the need for genome editing as may be required in a true autologous transplant (see above).

It is important to point out that while such a model seems attractive as compared to a truly autologous model it is still not as cheap as having a allogeneic therapy where a single or two or three cell lines are selected for their ability to grow and differentiate into the required end product which can be used for therapy. Proponents of such allogeneic therapy model have argued that immune suppression may not be required in many cases such as when cells are only required for a short time period or when cells themselves are not immunogenic or when cells are transplanted into immune privileged sites [11–13]. Investigators have noted as well that in the case where immune suppression is required localized immune suppression may be effective and in any case current matching and the presence of minor antigen mismatches can be sufficient for rejection whether the major antigens are matched or not matched. The cost benefit and potential utility may make these the preferred choice for some PSC based therapy.

Overall it appears to us that each approach may be uniquely suited to certain disease indications. For cardiac transplant in congestive cardiac failure or in pediatric malformations or for bone defect repair sufficient time is available to obtain autologous cells, differentiate them and then transplant them. Lung, gut, liver and spleen are generally thought to be more immunogeneic and in acute disorders of such organs allogeneic therapy is unlikely to work but HLA matched cells with immune suppression may be a viable choice. Likewise in mongeneic diseases where gene editing is difficult but could be standardized (such as replacing a whole exon rather than correcting a point mutation) or using a safe harbor strategy one can imagine that the economies of scale and time saving would make banked types cells a cell of choice even in diseases with a slow progression. Allogeneic therapy may be an ideal cost effective solution where cells are required for immune stimulation over a short time period or to provide cells with a short life cycle or when cells are being transplanted into immune privileged sites such as what has being attempted in Parkinson`s disease [14, 15]. In the subsequent sections we review the process of immune matching, the process of rejection and possible alternatives to a HLA bank.

## Transplant Rejection and the Immune System

The rejection response to grafted tissue is caused by cell surface molecules that induce an antigenic stimulus (see Table 1). The immune system fully matures during early fetal development and an immune response to self antigens is extinguished through a careful processing step in the thymus and spleen. This allows one to distinguish self-antigens from non-self and is the basis for successful transplantation [16–19]. Thus delivery of cells prior to this maturation is successful even if no matching is performed and even xenotransplants can be tolerated and tolerization strategies can be developed [20–24]. However, it is important to remember that only self-antigens that are present at this stage are recognized as self. Embryonic proteins that are not expressed at this stage will be rejected and nuclear and cytoplasmic antigens that are not presented to the thymus can still generate an immune response as is seen in certain chronic diseases. These have important implications when considering IPSC based therapy. IPSCs may be immature and even their differentiated products may express embryonic antigens to which an immune response may be mounted. Likewise it may be possible to treat individuals at an early stage with allogeneic cells with immune suppression. Immunologists have also learnt that one can tolerize individuals to antigens so that certain mismatches can be tolerated. Perhaps the best example of this is tolerization to ABO mismatches in kidney transplants [23, 24] (Table 2).

**Table 1:** MHC based cell rejection. The table briefly summarizes the issues related to cell transplants being rejected. The MHC systems is primarily responsible for recognizing self vs non-self. However other antigens and the innate immune system also contribute to rejection

The MHC system & foreign antigens
• MHC Class I by most cells in adult Including neural stem cells.
• Embryonic cells have little or none but will express them on inflammation or differentiation
• MHC Class II by professional APC such as T cells, B cells, macrophages, endothelial cells and thymic epithelial cells
• Different HLA antigens responsible for rejection at different time points. HLA-DR mismatch important in the first 6 months, the HLA-B in the first 2 years, and HLA- A mismatches over the long-term
• Foreign antigens are presented by cells expressing Class I or II peptides on surface and lead to activation of T cells, B cells and macrophages.
• ABO blood groups and sex differences may have effects on transplants
• T–regs, Complement, atypical MHC antigens (HLA G), minor antigens and modulators of local immune response (indoleamine, NO, etc.) can exacerbate or inhibit rejection
• GVH (graft versus Host) immune issues may be important for blood derivatives

**Table 2:** Reduced immunogenicity of cells. The table summarizes the likely reasons cells may be less immunogenic than tissues or organs or marrow. In bold we list the reasons why cell based therapy may be more immunogenic. The pathways for rejection are summarized in the first column to remind the non expert as to the different pathways that are activated in rejection

Rejection	PSC based cell transplant should be less immunogenic
• ABO blood group mediated	• Presumably no DC cells in most transplants
• Complement mediated	• Cell likely transplanted to immune privileged sites
• Adaptive immunity	• No ABO antigen response in most cases
• Innate immunity	• IPSC/ESC cells may have some tolerance themselves and MSC and other stem cells maybe immune modulatory
• Graft versus Host	• No vasculature or complement mediated rejection mechanisms
• Male vs. female	• Other antigen presenting cells (support cells) may not be present in the transplant

But: Fetal antigens may be present, Foreign protein may be present from culture, atypical antigens may be formed, silenced genes expressed, unknown therapeutic proteins may be present which may create rejection

While there are exceptions as we have described above nevertheless in the vast majority of cases when non-self tissue is transplanted it evokes an immune response which is mediated by a wide variety of transplantation antigens have been described, including the MHC molecules, minor histocompatibility antigens, ABO blood group antigens, and monocytes/endothelial cell antigens (Table 2). The minor histocompatibility antigens are processed peptides derived from cellular antigens that are presented by MHC molecules but are not derived from the MHC. ABO incompatibility can result in hyperacute rejection of primarily vascularized grafts, such as kidney and heart and could be an issue for IPSC derived hematopoietic products [25–27]. Rejection is not always acute but may be chronic and indeed different HLA antigens appear to mediate different aspects of rejection Experience in transplantation immunology has led to the realization that the major impact in graft loss comes from the effects of HLA-B and -DR antigens. There also appears to be a temporal HLA mismatching effect. HLA-DR mismatch is most important in the first 6 months after transplantation, the HLA-B effect emerges in the first 2 years, and HLA-A mismatches have a deleterious effect on long-term graft survival [28–30]. In general a primary rejection response sensitizes the recipient and the second exposure to the same antigen(s) results in a greater, more rapid response that leads to rejection [31]. Cross sensitization can occur as well and activation of the immune system by non specific stimuli can override local inhibitory effects and lead to rejection of otherwise tolerated mismatches [32]. This is an important consideration when one considers therapy that may require repeated transplants. It is also important to note that different regions of the body show varying degrees of rejection and in certain specialized instances (such as pregnancy and cancer) mismatched alleles can be tolerated [33].

An additional consideration that is of importance in transplants related to the hematopoietic system is the issue of graft versus host disease [34, 35]. Immune cells present in the transplant can recognize recipient antigens as foreign and activate an immune response by mobilizing host macrophages and other effector cells. While this may not be relevant in most PSC based therapy this will become of importance in delivery of PSC derived cells of the hematopoietic lineage.

Thus PSC derived cells may provoke both an innate and acquired immune response to embryonic antigens, foreign proteins that are carried over from the culture system, minor HLA antigen incompatibility, major HLA incompatibility and the severity of the response depends on where the cells are transplanted, the immune status of the individual and whether the transplant is the first or one in a series of such transplants and whether the individual has been cross sensitized by exposure to other antigens. Rejection processes are similar to those of organs and bone marrow though likely to be less severe (see below).

## Immune Suppression Regimes after HLA Matching

While searching for an unrelated donor, high-resolution (4-digit) genetic typing of both the patient and the donor is necessary. The current standard of HLA typing has evolved from bone marrow, cord blood and organ transplants is typing at the HLA-A, HLA-B, HLA-C, HLA-DRB1, and HLA-DQB1 genetic loci. An hematopoietic stem cell transplant (HSCT)donor is referred to as a “10/10 allele match” or “perfect match” when both HLA alleles are identical at each of the HLA-A, HLA-B, HLA-C, HLA-DRB1, and HLA-DQB1 loci. A similar HLA matching model is used for organ transplants [36–39].

It is important to understand that even with a perfect match between unrelated donors minor histocompatibility antigens, which are naturally processed peptides derived from normal cellular proteins, may evoke a strong MHC-restricted response because of the presence of different polymorphisms in the donor and in the recipient (Tables 1 and 3). Likewise the innate immune response may precipitate a reduction in function of the graft. Natural killer (NK) cells may also contribute to alloreactivity, particularly in haploidentical HSCT, through an interaction between killer immunoglobulin-like receptors (KIRs) on NK cells and HLA class I alleles (particularly HLA-C) on mismatched cells. As a consequence of this biological reality even in fully matched donations from unrelated donors immune suppression regimes are considered necessary. In bone marrow transplants these are done for a lifetime. It is likewise considered necessary in transplants of highly vascularized tissue such as liver, heart or pancreas. Indeed, it was the breakthroughs in immune suppression such as cyclosporine treatment, CTL antibodies and antithymoglobin which allowed for the development of bone marrow and solid organ transplants. The average cost of such immunotherapy is approximately $20 K/year and the immune suppression therapy has some morbidity as well [40]. Several investigators have tired to eliminate or reduce the immune regimes 1–2 years after transplant with mixed results [41, 42].

**Table 3:** HLA typing and immune suppression. As discussed in the text cells may be less immunogeneic than tissues or organs and this has raised that possibility that with HLA matching no immune suppression will be required. The argument for and against this are summarized. For a detailed discussion the reader is directed to the references that discuss these issues in detail

Will HLA typing allow us to eliminate use of immunosuppressants?	
YES– Because	No– Because
• Cells are less immunogenic than organs or tissue	• But nevertheless cells are immunogenic
• Data that immune suppression can be removed	• However in kidney and islets these data are controversial
• Data that fetal cells can tolerate some degree of mismatch	• True but only limited mismatch tolerated
• Many target therapies are in immune privileged sites	• However, blood and other non immune privileged sites being considered
• Embryonic cells have low or no MHC expression	• Embryonic cells will elevate expression after transplantation and in cases of sensitization or immune activation
• Cells may have localized immune modulatory activity	• True but this may be overcome when homeostasis changes

If foreign protein expressed then immune suppression to that antigen will be required

Initially there were concerns that iPSCs (and ESCs) unlike other cells may be more immunogenic [43] than organs and marrow and even other cells or that neoantigens may be present [44, 45]. More recent work by Guha, Morizane and others [46–48] provided data that autologous iPSC-derived cells may not be more immunogenic. Indeed, It has been shown that mouse ES cell-derived tissues display an inherent capacity for immune privilege which permits the acceptance of tissues across an MHC barrier without recourse to any form of immune intervention [49, 50].

On the other hand, iPSCs, in contrary to ESCs, may have residual information from the cell of origin, a phenomenon known as epigenetic memory. The effects of epigenetic memory have just started to be explored. However, recent work suggested that neurons derived from reprogrammed fibroblasts are more immunogenic than those derived from mesenchymal stromal cells [51]. It is also important to note that epigenetic memory seems to be lost during successive passages in culture [52]. Irrespective of the functional relevance of this phenomenon, we believe that this iPSC-specific issue should not be overlooked without thorough discussion, in particular in the case of iPSC-derived, clinical-grade cells to be transplanted.

It is now thought that cell transplants will require less immune suppression particularly for tissues that do not contain MHC class I antigen presenting cells such as transplanting neurons in the CNS. Likewise transplants with stem cells which express low or no HLA antigens may require limited immune suppression or where localized immune modulation is present [53–55] Indeed, cells expressing no HLA antigens, obtained by gene editing have been proposed as a future therapeutic alternative that can by-pass immune surveillance and therefore will eliminate the need for immunosuppression. Recently, HLA-A null cells have been obtained [56]. On the other hand, transplanting cells that will not be recognized by the immune system possesses intrinsic risks, that are currently being discussed. Similarly transplants of the cornea, in transplants into tissue where vascularity is limited, mismatched grafts appear to be well tolerated and indeed the anterior chamber of the eye has been used effectively as an immune privileged site even for xenotransplants. The eye, like the brain is considered immune privileged, owing to BBB properties as well as other active cell mediated interactions known as anterior chamber-associated immune deviation [ACAID]. Using cord blood cells has provided evidence that a 1 or 2 locus mismatch can be tolerated from such cells [54, 55] and raised the hope that this may be the case for other developmentally immature  immature cells.

## Transplants into the Central Nervous System

Besides the presence of the BBB, the immune response in the central nervous system differs from other tissues and organs mainly due to the absence of dendritic cells, conventional lymphatics, the downregulation of major histocompatibility complex (MHC) molecules within the CNS parenchyma, and the presence of local immunosuppressive factors [57–59]. The immunomodulatory effects of astrocytes in the CNS could further contribute to the immune-privileged state [54]. To date, the main areas of allogenic cellular therapeutic transplantation strategies targeting brain and spinal cord disease or injury have included human fetal dopaminergic cells (Parkinson’s Disease (PD)), human fetal neural stem cells (Pelizaeas Merzbacher disease (PMD), spinal cord injury (SCI) and amyotrophic lateral sclerosis), and human ESC derived oligondendrocyte precursors (SCI). In most cases a transient (60 days–12 months depending on the study) immune suppression strategy has been employed and graft persistence has been documented up to 16 years post transplantation. Experience with CNS cellular transplants has thus confirmed that the immune response is modest and that this modest response could be readily attenuated when immunosuppression was used during the initial period of surgical breach of the BBB [60, 61]. These results largely confirmed the assumptions based on rodent syngenic, allogeneic and autologous transplants that had been performed in the 1990’s [62] In addition, host immune system monitoring has failed to detect graft directed immune responses. Similar data with mismatched or allogeneic MSC suggest that MSC while not immune privileged are immunomodulatory and do not provoke a large rejection response and in many instances can tamp down a immune response [63–65].

While data for PSC-derived cell  transplants in humans is limited the results so far suggest that immune suppression is likely required but may be less that what is necessary for organ or marrow transplants (Table 2). Clinical trials using fetal tissue transplantation into the brain of PD patients have used long-term, short-term or no immunosuppression [66]. In some cases, it has been argued that stopping immunosuppression had detrimental consequences to the survival, growth or function of the transplanted cells [66]. In addition, inflammatory signals have been shown to affect the survival, differentiation and proliferation of neural  progenitor cells in animal models [67]. Therefore, we would argue that one should be cautious in assuming that no immune suppression will be required over the long term as cells may begin to express immunogenic antigens or the immune system begin to react to antigens present on these cells in response to other insults or exposure to infections as may occur years or decades after a transplant. Importantly, peripheral, sustained inflammation can modulate brain inflammation and exacerbate neurodegeneration in the substantia nigra in animal models, increasing the complexity of the analysis of the possible consequences of immunomodulation on PD progression [68]. In summary despite the evidence that cells themselves may be less immunogenic than solid organs and bone marrow it is also clear that transplanted cells can be rejected and thus one would prefer using HLA matched cells rather than mismatched cells should such cells be available. iPSC technology allows one to consider creating such a bank and several issue related to generating such a bank are under active discussion. In the next section we summarize some of the considerations in developing such a bank.

## Size of Bank and its Creation

HLA are highly polymorphic, and gene sequencing analysis and more than 2558 HLA class I and II alleles have been recognized. This variability would suggest that the number of lines required would be a daunting task. However, HLA antigens are inherited in a Mendelian dominant manner. HLA genes are almost always inherited together, thus the antigens of the entire HLA region inherited from one parent collectively are called haplotype. In humans, these genes reside in the short arm of chromosome 6. Because chromosome 6 is an autosome (a chromosome with two pairs), all individuals have two HLA haplotypes (one for each chromosome). According to this, any sibling pair has a 25 % chance of inheriting the same two parental haplotypes, a 50 % chance of sharing one haplotype, and a 25 % chance of having two completely different haplotypes. All children are haploidentical with each parent. Based on these facts several empirical calculations can be made based on frequency, ethnic diversity and the type of therapy that will be required. Several groups have performed such calculations [69–76]. According to one estimate, an iPSC bank from 150 selected homozygous HLA-typed volunteers could match 93 % of the United Kingdom population with a minimal requirement for immunosuppression [74]. Similarly, as few as 50 such lines could potentially match 90% of the Japanese population [69].  A similar limited number of lines may be sufficient for Korea and the Han population in China and perhaps Argentina (77–81 and references therein and C. Gamba, personal communication).  However, more diverse populations will require more lines as would be expected for Brazil, US and India (81 and references therein). The data therefore suggest that creation of a “haplobank” of iPSC lines homozygous for a range of HLA types representative of different geographical populations and ethnic groups could simplify HLA matching, provide matches for a reasonable percentage of a target population, and extend iPSC-derived therapies beyond the autologous setting.

**Fig. 1:**
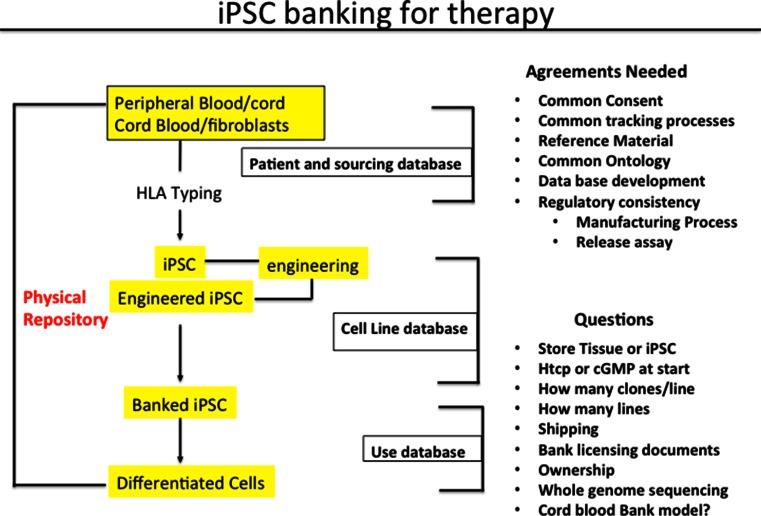
Making iPSC banks. A flowchart of the IPSC banking process is provided. Note that three types of data bases will be required and that there are important decisions that need to be made at each step. No clarity or previous guidance from the regulatory authorities exist. Decisions will need to be made early to avoid the cost of recreating banks which have the potential of lasting over decades

Although only a limited number of lines are required it will be critical to identify the right donor to generate lines that carries the representative Haplotype. Thus one needs a source of clinically compliant tissue sample which is typed for HLA and access to the data set to select individual donors that are most representative as contributors to a bank. The steps an issues that need to be considered are summarized in Fig. 1. Of the various questions perhaps the most important one is what starting sample to use and how one may harmonize collection and storage and distribution of lines across the world. These issues and concerns are being debated currently and the reader is directed to those articles for a detailed discussion [82, 83].

## Strategies to Extend the Utility of a Haplobank

Although banking of HLA matched samples is one possible solution to resolving immune issues there are several other potential strategies that have been considered. We describe some of them and suggest that application of these strategies can complement the Haplobank approach and in some cases may render the use of immune matched cells unnecessary. Some of these approaches are summarized in Table 4.

**Table 4:** Additional ways to modulate the immune system. The table summarizes the various strategies that have been proposed to reduce or eliminate a immune rejection of cells. Some of these strategies are only possible with pluripotent cells and some require extensive gene engineering. For detail see text and references

Ensuring survival of transplanted cells
• Autologous or Syngeneic transplant
• Transplant in utero
• Transplant to an immune privileged site
• Co-transplant of immune modulatory cells
• Localized immunesuppression (Indoleamine, NO, HLA-G)
• CTL blockade, anti-TCR therapy
• ABO tolerization and other antigen tolerization
• T-regs- (CD24-) to induce tolerization
• Mixed chimerism with accompanied bone marrow transplant
• iPSC engineering
• Generation of DC cells
• Thymic rejuvenation
• HLA matching with immune suppression

Perhaps the simplest approach based on findings describe above with transplants of fetal cells is to consider localized immune suppression. Successful local suppression with long term engraftment has been described and is based on the pathways that enable a mismatched fetus to survive for prolonged periods as a transplant with a shared circulation. These include molecules such as indolemine, nitric oxide synthase and prostaglandins that modulate macrophage and microglial behavior [84]. More recently it has been suggested that some stem cells possess such localized immune modulatory activity and cells such as mesenchymal stem cells (MSC) especially placental derived MSC may have a capability along these lines. Therapy may then consist of co-transplantation of the functionally required type with mixed with MSC or cells engineered to express localized immune modualtory genes (see references above).

Several other strategies have been proposed [85–95]. One solution is to take advantage of the pluripotency of iPSCs to generate not only therapeutic cells but also immature cells of the immune system such as dendritic cells expressing neoantigens to which tolerance is required [88] . Another critical area of investigation into strategies to induce donor-specific tolerance is rejuvenation of the thymus [89–91]. The thymus is the main organ responsible for establishing immune tolerance via elimination of autoreactive T cells. iPSCs could be used as a replacement thymic epithelial cells (TECs) that could be used to induce tolerance to an iPSC-derived graft. Two recent studies describe progress in generating TECs from human PSCs, although work remains to be done improve their maturity and functionality [89–91]. Other approaches include development of immune-privileged PSC derivatives capable of blocking the activation of co-stimulatory receptors responsible for immune recognition. This could be accomplished by genetic “knock-in” of ligands of potent inhibitory receptors expressed by T cells (e.g. CTLA4 or PD-1) or by targeting inhibitory pathways that mediate immunosuppression (e.g. indoleamine 2,3-dioxyge-nase or HLA-G) [92–94]. Likewise monoclonal antibodies could be used. For example Pearl et al. [86] showed that monoclonal antibody-mediated co-stimulation/adhesion blockade of host T cells can result in long-term engraftment of hESC and human iPSC grafts in murine models.

Thus the haplobank could serve not only as a source of partially immune matched cells but could also be as a source of transplants of mismatched cells by permitting the standardized generation of tolerization inducing cells such as dendritic cells or thymic epithelial cells. Likewise having a bank of well characterized cells with appropriate patient history collected in a clinically compliant fashion would allow gene engineering to effectively modulate an immune response (56). Such gene engineering strategies have been shown to be efficient and reliable (56).  The utility of the bank could thus be further extended and the use of long term immunosupressive drugs could be further reduced.

## Summary

Several strategies can be utilized to overcome the rejection of mismatched cells. At one extreme one can generate autologous cells and on the other one can use mismatched cells that have been modified or are co-transplanted with reagents that allow such cells to bypass the rejection phenomenon.. A intermediate approach is to reduce the rejection possibility by using HLA matched cells followed by limited immunosuppression as dictated by the cell type delivered and the host immune status. No solution seems to be clearly superior over another and cost, regulations and scientific breakthroughs will determine which strategy will be the strategy of choice in the future. iPSC clearly have the potential to reduce the cost of cell based therapy and likely will play an important role in an future approach to personalized medicine.
